# Developing a carotid ultrasound radiomics-semantic fusion model to identify aortic dissection: a two-center retrospective study

**DOI:** 10.3389/fmed.2026.1813428

**Published:** 2026-04-29

**Authors:** Yan Cui, Hui Wang, Jun Wu

**Affiliations:** 1 Department of Cardiovascular Ultrasound, Second Affiliated Hospital of Dalian Medical University, Dalian, Liaoning, China; 2 Department of Ultrasound, First Affiliated Hospital of Dalian Medical University, Dalian, Liaoning, China

**Keywords:** aortic dissection, carotid ultrasound, machine learning, nomogram, radiomics

## Abstract

**Background:**

Aortic dissection (AD) is a life-threatening cardiovascular emergency. Preventive strategies with proven efficacy remain limited, and early identification and timely diagnostic workup are critical. This study aimed to develop and externally validate a fusion model integrating carotid ultrasound radiomic features with clinical and ultrasound semantic variables to discriminate AD from non-AD participants in a retrospective case–control setting.

**Methods:**

We retrospectively enrolled 209 participants and randomly allocated them to training and test cohorts in a 7:3 ratio. Additionally, we selected 39 external participants as the validation set. We developed three diagnostic models: a carotid artery ultrasound radiomic model, a semantic model, and a fusion model combining both approaches. Subsequently, we compared the diagnostic efficacy of the three models.

**Results:**

The area under the curve (AUC) metrics for the semantic, radiomic, and fusion models demonstrated values of 0.73, 0.84, and 0.94, respectively, in the training set, with corresponding values of 0.73, 0.87, and 0.93 in the test set. In the external validation set, the AUCs of the three models were 0.71, 0.81, and 0.91, respectively. Statistical analysis revealed significant differences in discriminative performance between the fusion model and other models (*p* < 0.05). Furthermore, the fusion model exhibited superior performance across multiple evaluation metrics, including accuracy, sensitivity, F1 score, calibration metrics, and clinical decision curve analysis.

**Conclusion:**

Carotid ultrasound radiomics and semantic features showed diagnostic value for distinguishing AD from non-AD participants. The fusion model further improved discriminative performance, supporting future prospective multicenter studies to evaluate clinical utility in real-world triage workflows.

## Introduction

Aortic dissection constitutes a critical cardiovascular emergency characterized by a life-threatening hemorrhagic separation of the aortic media layer (1), often associated with major risk factors such as systemic hypertension, atherosclerotic vascular disease, and genetic connective tissue disorders (2). The condition manifests suddenly, with hemodynamic instability and a high mortality rate. Epidemiological estimates suggest that approximately half of affected individuals succumb before hospital admission, complicating precise incidence calculations (3). Current data indicate an annual global incidence of approximately 2 to 16 cases per 100,000 population (4). Studies reveal that Stanford Type A aortic dissections carry a 20–30% mortality rate within the first 24 h, with mortality increasing by 1–2% per hour subsequently, reaching 50% at 48 h. For patients deemed inoperable, 30-day or in-hospital mortality rates range from 50 to 79% (5). The exact pathogenic mechanisms underlying aortic dissection are not fully elucidated. Surgical repair remains the definitive treatment modality, as pharmacologic options with proven efficacy are limited. Consequently, early identification of high-risk cohorts, implementation of secondary prevention strategies, and prompt surgical intervention are essential for optimizing patient prognosis.

The carotid artery is an elastic artery that shares similar histological and physiological characteristics with the aorta, coronary arteries, and iliac arteries (6). It withstands relatively high vascular wall stress, and its hemodynamic profile closely resembles that of major central arteries such as the aorta and coronary arteries. Consequently, the carotid artery functions as a vascular window, reflecting the hemodynamic and stiffness properties of other large elastic arteries throughout the body (7, 8). Due to its superficial location, it is easily accessible for high-resolution, non-invasive imaging techniques, particularly ultrasound, enabling clear and precise assessment. Numerous studies have utilized carotid ultrasound to evaluate the extent and severity of atherosclerotic lesions and to monitor the efficacy of lipid-lowering therapies and surgical interventions (9–13). The pathophysiological mechanisms underlying carotid atherosclerosis are highly analogous to those observed in systemic large-vessel atherosclerotic disease (14). Therefore, monitoring the carotid artery provides insight into the degree and severity of atherosclerotic lesions in other major vessels, including the aorta. Furthermore, research indicates that core pathological features of carotid plaques—such as large lipid necrotic cores, thin fibrous caps, intra-plaque hemorrhage, and prominent inflammatory infiltration—are closely associated with plaque vulnerability (15, 16). These features can be identified through characteristic ultrasonographic signs, highlighting the importance of high-resolution imaging in assessing plaque stability (17).

Nevertheless, conventional sonographic imaging predominantly depends on operator-dependent qualitative interpretations and subjective morphological descriptions, which lack standardized quantitative parameters. This approach not only generates inter-reader inconsistencies but also limits the detection of subtle morphological abnormalities that may escape visual recognition. There exists a pressing clinical imperative to derive comprehensive quantitative imaging biomarkers from large-scale diagnostic datasets. Radiomics, as an evolving multidisciplinary methodology, encompasses the extraction of non-invasive, high-dimensional quantitative parameters from complete lesional volumes and performs comprehensive phenotypic imaging analysis to improve diagnostic precision in complex conditions (18, 19). This investigation seeks to develop an interpretable imaging-clinical fusion model based on carotid ultrasound radiomics to aid in identifying AD (vs. non-AD) and prioritizing further diagnostic workup in clinically relevant settings.

## Materials and methods

### Participants

**Figure 1:**
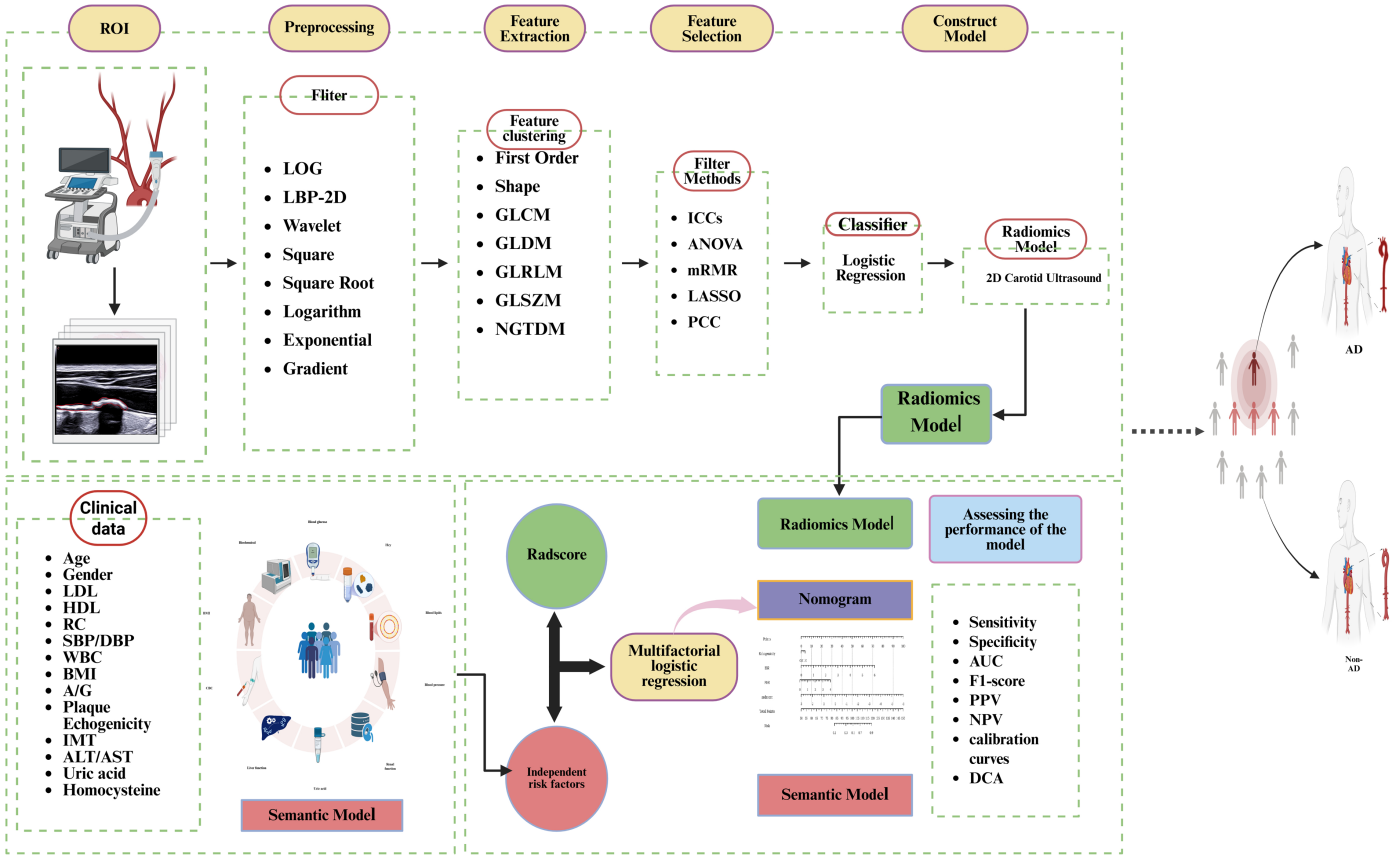
The flow chart of the study design. Created with BioRender.com.

This study was approved by the Medical Ethics Committee of the First Affiliated Hospital of Dalian Medical University as well as Dalian Jinzhou District People’s Hospital. All data were de-identified to protect patient confidentiality. We conducted a retrospective case–control study including patients with computed tomography angiography (CTA)-confirmed aortic dissection (AD) and controls without AD who presented to our institution between January 2022 and January 2025. Controls were defined as individuals without AD based on CTA whenever available; when CTA was not clinically indicated, alternative imaging such as magnetic resonance angiography (MRA) or transesophageal echocardiography (TEE) was accepted as the reference to exclude AD. We also collected an external validation cohort of eligible participants from Jinzhou District People’s Hospital during the same period. Collected variables included demographics (age, sex), body mass index (BMI), systolic/diastolic blood pressure (SBP/DBP), and laboratory biomarkers (white blood cell count, lymphocyte count, blood glucose, high-density lipoprotein (HDL), low-density lipoprotein (LDL), total cholesterol (TC), remnant cholesterol (RC), uric acid, creatinine, alanine/aspartate aminotransferase (ALT/AST), homocysteine, and albumin/globulin ratio (A/G)), as well as standardized 2D carotid ultrasound images. Missingness for each variable was <10%; continuous variables were imputed using the mean (approximately normal) or median (skewed), and categorical variables using the mode, based on the training set only. The fitted imputation parameters were then applied unchanged to the test and external cohorts. Missingness proportions are reported in Supplementary Tables S1, S2. Inclusion criteria: (1) The case group recruited patients with a definitive diagnosis of AD confirmed by computed tomography angiography (CTA). (2) The control group comprised individuals in whom aortic dissection was systematically excluded by evaluation with CTA, magnetic resonance angiography (MRA), or transesophageal echocardiography (TEE). (3) For all participants, clinical data collection and standardized imaging examinations were completed within a uniformly predefined time window, and complete, traceable clinical and imaging datasets were available. (4) Imaging quality met diagnostic requirements and allowed for accurate segmentation of regions of interest (ROI), as well as subsequent extraction and analysis of radiomic features. Exclusion criteria included: (1) Patients who have not undergone standardized carotid ultrasound examination, resulting in a lack of key vascular structural and hemodynamic data. (2) Imaging studies with significant artifact, motion interference, or inconsistent scanning parameters, rendering them non-diagnostic for analysis. (3) Cases with extensive missing data for critical clinical indicators, where reasonable imputation or statistical inference using historical or follow-up data is deemed unfeasible. The study workflow is illustrated in Figure 1.

### Clinical characteristics and ultrasound image collection

For each participant, an index time was defined as the emergency admission time for acute presentations or the outpatient evaluation date for non-acute visits. For AD cases, laboratory tests were obtained within 12 h and carotid ultrasound within 24 h of the index time whenever available; for non-AD controls, laboratory tests and ultrasound were obtained during the same clinical encounter. Because clinical pathways and available timestamps differed between cases and controls, residual time-window confounding cannot be fully excluded and is discussed as a limitation.

Carotid 2D ultrasound examinations were performed using a Philips EPIQ-7 ultrasound system with an L12-5 linear-array transducer (5–12 MHz). Participants were scanned in the supine position with the head turned contralaterally. Standardized images were acquired at predefined segments, including the distal common carotid artery (approximately 1–2 cm proximal to the bifurcation) and the carotid bulb, in both longitudinal and transverse planes. Acquisition parameters (depth, gain, time-gain compensation, dynamic range, focal zone) were adjusted to achieve an anechoic lumen and clear delineation of the intima–media interfaces and plaque boundaries. Typical depth settings ranged from 3.5 to 4.5 cm and were adjusted based on patient habitus. Images were exported in DICOM format without additional post-processing whenever possible. Both centers followed the same acquisition protocol to maintain image integrity.

Two certified vascular sonographers with over 8 years of carotid duplex ultrasonography expertise independently analyzed and documented each participant’s clinical parameters (age, sex, BMI, SBP, and DBP) and laboratory indicators (WBC, HDL, LDL, TC, RC, serum uric acid, serum creatinine, ALT/AST, A/G, blood glucose, and homocysteine) while blinded to group assignment. The 2D carotid ultrasound images were acquired and re-evaluated, with ultrasound reports detailing intima-media thickness, intima-media surface smoothness, plaque echogenicity classification (hypoechoic, predominantly hypoechoic mixed, predominantly hyperechoic mixed, hyperechoic), plaque multiplicity, and surface morphology. Any discrepancies in interpretation were adjudicated by a senior radiologist with over 20 years of experience in vascular ultrasonography. Notably, after curating a cohort of patients diagnosed with AD, adhering strictly to predefined inclusion and exclusion criteria, we subsequently recruited individuals of Non-AD, who were propensity-matched based on sex, age, and BMI.

### Image segmentation

All 2D carotid ultrasound images were uploaded to the Darwin research platform for ROI annotation and radiomic feature extraction. Using stratified sampling by outcome label, 209 participants were randomly split into a training set (*n* = 146) and a test set (*n* = 63) at a 7:3 ratio. An independent external validation cohort (*n* = 39) from Jinzhou District People’s Hospital was analyzed separately. ROI definition: bilateral carotid arteries were scanned. For radiomics analysis, a single representative image was selected from the side showing the most pronounced atherosclerotic change (largest plaque area, or the greatest intima–media thickening/irregularity when no plaque was present). If both sides were comparable, the right side was selected. When plaques were present, the ROI encompassed the entire plaque on the frame with maximal plaque area, excluding the lumen and acoustic shadow. In the absence of plaque, the ROI was defined as a 1-cm segment of the distal common carotid artery immediately proximal to the bifurcation, including the intima and media layers. For multiple plaques, the largest plaque was selected. All segmentations were performed by trained readers blinded to outcomes.

### Radiomics feature selection and Radscore calculation

We uploaded the two-dimensional carotid ultrasound images of 209 patients to the Darwin Scientific Platform for radiomic feature extraction. Utilizing the open-source toolkit PyRadiomics, the platform performed the quantitative computation of the medical imaging features. Based on the feature classification criteria specified in the official Pyradiomics documentation^1^, radiomic features were divided into first-order statistical features, second-order textural features, and higher-order statistical features, which can be further categorized into seven major categories: (1) First-Order Statistics; (2) 2D Shape features; (3) Gray Level Co-occurrence Matrix (GLCM); (4) Gray Level Dependence Matrix (GLDM); (5) Gray Level Size Zone Matrix (GLSZM); (6) Neighboring Gray Tone Difference Matrix (NGTDM); and (7) Gray Level Run Length Matrix (GLRLM). To augment feature dimensionality and strengthen image characterization capability, eight distinct image filters were employed in this study to perform transformation on the original images, including Laplacian of Gaussian (LoG), Local Binary Pattern (LBP), Wavelet Transform, Square Transform, Square Root Transform, Logarithm Transform, Exponential Transform, and Gradient Transform. First-order statistical features and gray-level textural features were subsequently extracted from these filtered images. In total, 1,125 radiomic features were ultimately extracted from the carotid artery 2D ultrasound image.

During the feature selection phase, min-max normalization was first applied to the raw feature vectors, linearly scaling each feature to the [0, 1] range to hasten model convergence and enhance modeling stability. Intra-observer and inter-observer reproducibility assessments were conducted on extracted radiomic features. Specifically, two doctors were randomly selected for repeat segmentation. For intra-observer agreement, Reader 1 repeated ROI delineation after a 2-week washout period; for inter-observer agreement, Reader 2 independently delineated ROIs while blinded to outcomes. ICCs were calculated using a two-way random-effects model with absolute agreement and reported with 95% confidence intervals (CI), with features scoring below 0.75 excluded to ensure unstable radiomic features were removed. To further eliminate redundant and irrelevant features and build an efficient, robust predictive model, a multi-stage feature selection strategy was implemented. Initially, analysis of variance (ANOVA) was used to discard features showing a weak linear correlation with the classification label. Subsequently, the minimum redundancy maximum relevance (mRMR) algorithm was employed to compute mutual information among features and rank their importance, selecting a subset of features highly correlated with the outcome while minimizing inter-feature redundancy. Finally, the least absolute shrinkage and selection operator (LASSO) was used to perform further dimensionality reduction, preserving stable features with non-zero regression coefficients. Following a multi-step screening process, we narrowed 120 features down to 6. A radiomics score (Radscore) was then constructed by performing a weighted linear combination of these final features with their corresponding regression coefficients, calculated as: Radscore = β0 ± *Σ* (βi × xi). The specific software versions and key feature extraction parameters are detailed in the Supplementary Tables S9, S10.

### Model construction

To develop a clinically interpretable classifier, we constructed three models: (1) a carotid ultrasound radiomics model based on Radscore; (2) a semantic model using clinical and ultrasound semantic features; and (3) a fusion model combining Radscore with selected semantic predictors. The dependent variable was AD status (AD = 1, non-AD = 0). Candidate semantic predictors included ultrasound descriptors (plaque echogenicity, plaque number, IMT, plaque surface regularity, carotid intima–media surface regularity, and maximum plaque thickness) and clinical variables (age, sex, BMI, SBP, DBP, RC, WBC, blood glucose, and other laboratory markers). Categorical ultrasound descriptors were encoded using prespecified ordinal/binary schemes (e.g., plaque echogenicity treated as an ordinal variable from hypoechoic to hyperechoic). Variable selection followed a prespecified strategy within the training set.

### Statistical analysis

Continuous variables are presented as mean ± standard deviation or median as appropriate; categorical variables are presented as counts. Normality was assessed by the Shapiro–Wilk test. Between-group comparisons used Student’s *t*-test/ANOVA for normally distributed data or Mann–Whitney U tests otherwise; categorical variables were compared using chi-square or Fisher’s exact tests as appropriate. Model performance was assessed using the area under the receiver operating characteristic curve (AUC) and classification metrics including accuracy, sensitivity, specificity, and F1 score. For classification metrics, the operating threshold was determined in the training set by maximizing the Youden index (Youden = sensitivity + specificity − 1) and then applied unchanged to the test and external validation sets. AUCs were compared using the DeLong test. Calibration was assessed using calibration plots as well as calibration intercept/slope and the Brier score; uncertainty 95% CI for AUC and key metrics was estimated using bootstrapping. Decision curve analysis (DCA) quantified net benefit across threshold probabilities. Ten-fold cross-validation was used within the training data to assess model stability, and model selection (feature selection and tuning) was confined to the training data. All statistical analyses were conducted in R and relevant packages are listed in Supplementary materials.

## Results

### Reproducibility of ultrasound semantic measurements

The ICCs for ultrasound semantic measurements ranged from 0.89 to 0.96, indicating excellent intra- and inter-observer agreement (Supplementary Table S3).

### Participants’ demographic and clinical profile

A total of 209 participants were enrolled as the internal cohort, comprising 97 AD patients and 112 non-AD participants (including healthy individuals and patients with comorbidities such as hypertension, diabetes mellitus, and coronary artery disease). They were randomly divided into training and test sets at a 7:3 ratio, resulting in 146 and 63 participants in the training and test sets, respectively. The external validation cohort included 39 participants (18 AD patients and 21 non-AD participants). Baseline characteristics are summarized in Table 1 (internal cohort) and Supplementary Table S4 (external cohort), and cohort comparability across data splits is shown in Supplementary Table S5.

**Table 1:** Patients’ demographic and clinical characteristics table in the internal cohort.

Characteristic	AD (*n* = 97)	Non-AD (*n* = 112)	*p*
Age	61.32 ± 13.24	61.72 ± 14.86	0.91^a^
Gender			0.17^b^
Female	35	38	
Male	62	74	
BMI (Kg/m^2^)	26.05 ± 3.60	25.87 ± 3.75	0.66^a^
Plaque echogenicity			<0.001^b^
Hypoechoic plaque	38	7	
Predominantly hypoechoic heterogeneous plaque	39	28	
Predominantly hyperechoic heterogeneous plaque	18	42	
Hyperechoic plaque	2	35	
Number of plaques			0.79^b^
Solitary plaque	45	50	
Multiple plaques	52	62	
IMT (mm)	1.11 ± 0.25	1.15 ± 0.22	0.09^a^
Smoothness of plaque			0.61^b^
Smooth	41	45	
Non-smooth	56	67	
Smoothness of carotid inter-media			0.12^b^
Smooth	37	33	
Non-smooth	60	79	
Maximum plaque thickness	2.62 ± 0.87	2.24 ± 0.81	0.001^a^
Lymphocyte (×10^9^/L)	1.23 ± 0.60	1.63 ± 0.63	<0.001^a^
SBP (mmHg)	191.40 ± 25.18	155.67 ± 16.59	<0.001^a^
DBP (mmHg)	104.46 ± 18.82	85.46 ± 12.01	<0.001^a^
WBC (×10^9^/L)	11.03 ± 3.05	7.60 ± 2.91	<0.001^a^
HDL (mmol/L)	1.00 ± 0.24	1.08 ± 0.24	0.02^a^
LDL (mmol/L)	2.78 ± 0.81	2.69 ± 0.91	0.37^a^
TC (mmol/L)	4.73 ± 0.83	4.34 ± 1.00	0.03^a^
RC (mmol/L)	0.95 ± 0.30	0.57 ± 0.25	<0.001^a^
Uric acid (μmol/L)	338.12 ± 131.57	348.97 ± 130.91	0.53^a^
Blood glucose (mmol/L)	7.91 ± 2.54	6.52 ± 2.22	<0.001^a^
Creatinine (μmol/L)	75.50(34.75)	67.01(32.97)	0.19^a^
Homocysteine (μmol/L)	15.95 ± 9.54	14.08 ± 5.96	0.10^a^
ALT/AST	1.03 ± 0.41	0.99 ± 0.41	0.83^a^
A/G	1.24 ± 0.28	1.44 ± 0.33	<0.001^a^

a One-way ANOVA or Kruskal-Wallis test (as appropriate).

b Chi-square test or Fisher’s exact test (as appropriate).

AD means Aortic dissection, BMI means Body mass index, SBP means Systolic blood pressure, DBP means Diastolic blood pressure, WBC means White blood cell, HDL means High-density lipoprotein, LDL means Low-density lipoprotein, TC means Total cholesterol, RC means Remnant cholesterol, ALT means Alanine Aminotransferase, AST means Aspartate Aminotransferase, A/G means Albumin/Globulin Ratio.

### Building a semantic model

To mitigate overfitting and selection bias, the semantic (clinical-ultrasound feature) model was developed using multivariable logistic regression within the training set (20–22). Candidate predictors reflected routinely available clinical variables and ultrasound descriptors. Variable selection and encoding rules were prespecified, and multicollinearity was assessed using variance inflation factors. The final predictor list and regression results (coefficients/odds ratios with 95% CI) are provided in Supplementary Table S6 to enable independent replication.

### Establishment of radiomics model and fusion model

A total of 1,125 radiomic features were initially extracted from the 2D ultrasound ROIs. After reproducibility filtering (ICC) and redundancy reduction, 120 candidate features remained for subsequent selection. LASSO regression with 10-fold cross-validation selected six features with non-zero coefficients (Table 2). The Radscore was calculated as: Radscore = 0.059 + 0.838 × square_glcm_SumEntropy + 0.565 × exponential_glcm_JointEntropy + 0.351 × exponential_glcm_JointEnergy + 0.327 × exponential_gldm_DependenceEntropy − 0.304 × exponential_gldm_Dependence NonUniformityNormalized + 0.058 × square_glcm_JointEntropy. For readability, the suffix used in the Darwin/PyRadiomics export was omitted; a one-to-one mapping is provided in Supplementary Table S10.

**Table 2:** Definitions of the six selected radiomic features and their associations with AD.

Radiomics feature	Category	From	Significance	Correlation
SumEntropy	GLCM	2D images	A sum of neighborhood intensity value differences	+
JointEnergy	GLCM	2D images	A measure of homogeneous patterns in the image	+
JointEntropy (exponential)	GLCM	2D images	A measure of the randomness/variability in neighborhood intensity values.	+
JointEntropy (square)	GLCM	2D images	A measure of the randomness/variability in neighborhood intensity values.	+
DependenceNonUniformityNormalized	GLDM	2D images	A measures the similarity of dependence throughout the image.	−
DependenceEntropy	GLDM	2D images	A measure of the distribution of large dependencies.	+

**Figure 2:**
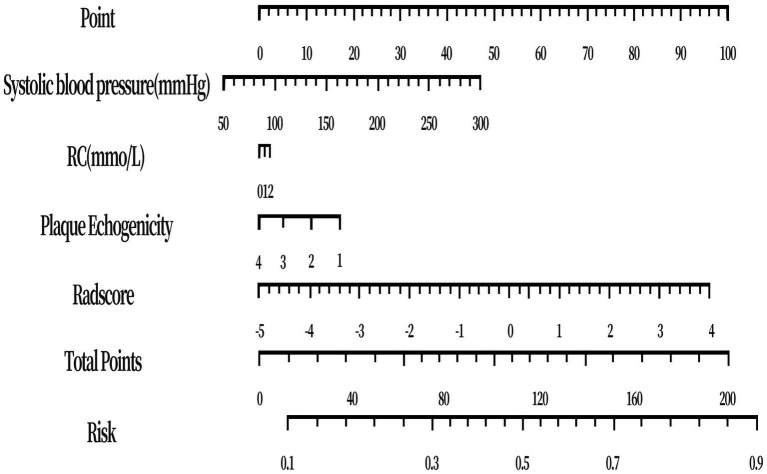
The nomogram of the fusion model. The visual nomogram comprises points, feature scales, total points, and predicted probability. In clinical use, practitioners assign points for each predictor, sum them to obtain the total points, and map the total points to the predicted probability of having AD at evaluation. For example, a patient with systolic blood pressure of 200 mmHg corresponds to 25 points; residual cholesterol (RC) of 1 mmol/L contributes 1.5 points; carotid plaque echogenicity classified as hypoechoic (coded as 1) adds 17 points; and a Radscore of 2 adds 74 points. The total is 117.5 points, corresponding to an estimated probability of approximately 0.52.

Supplementary Figure S1 illustrates the six radiomic features used to derive Radscore and their relative coefficients. To improve discriminative performance and clinical interpretability, we combined Radscore with key semantic predictors (plaque echogenicity, SBP, and RC) to build the fusion model and constructed a nomogram (Figure 2). The final multivariable logistic regression results (including regression coefficients and odds ratios) are reported in Supplementary Table S6.

### Evaluation of model efficacy

**Figure 3:**
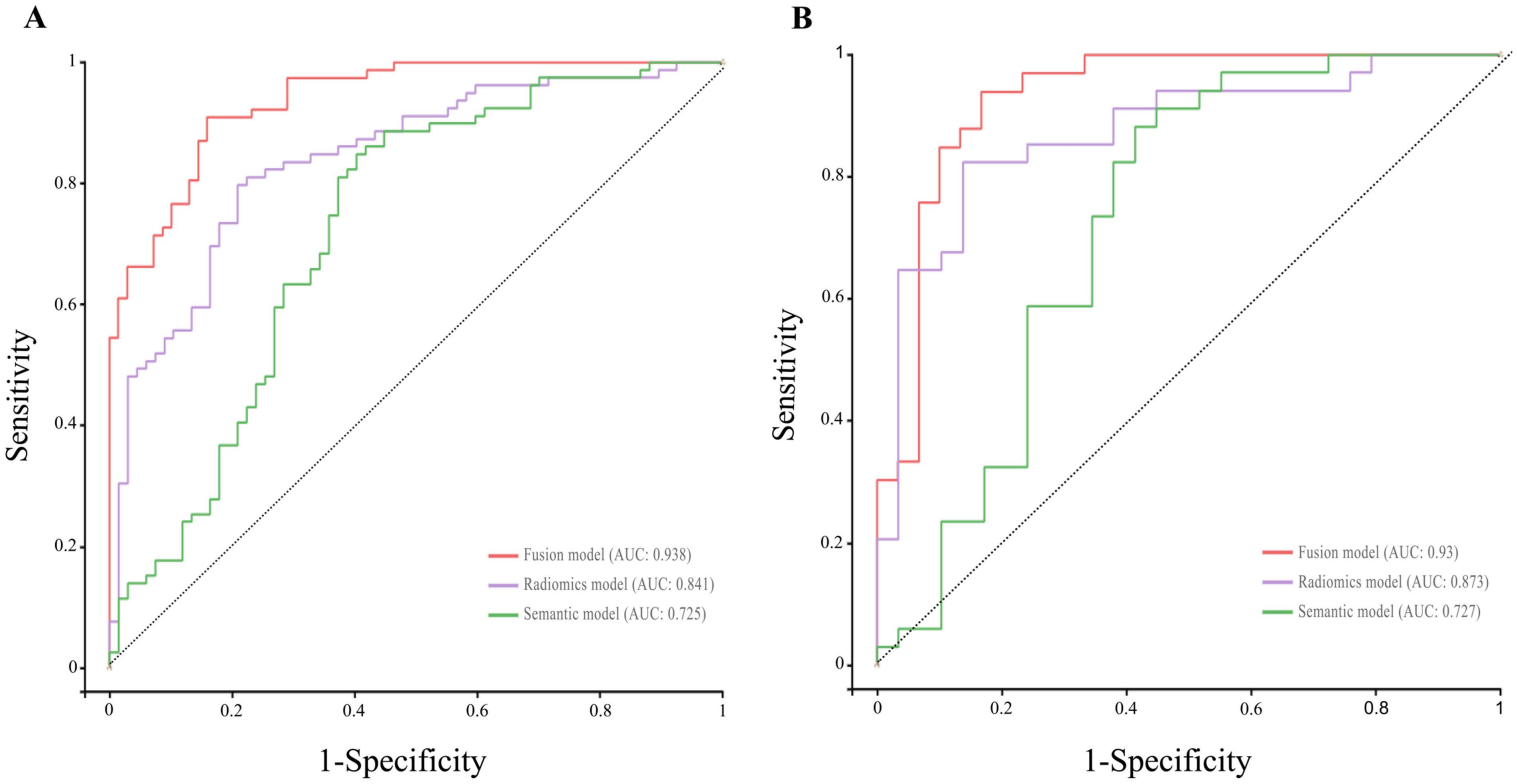
The receiver operating characteristic (ROC) curves for the three classification models. The receiver operating characteristic (ROC) curves for the three classification models in the training **(A)** and test set **(B)**. The horizontal axis represents 1 − specificity, and the vertical axis represents sensitivity. In the training set, the AUC values for the semantic model, radiomics model, and fusion model were 0.73 (95% CI: 0.64–0.81), 0.84 (95% CI: 0.78–0.91), and 0.94 (95% CI: 0.90–0.97), respectively. In the test cohort, the corresponding AUC values were 0.73 (95% CI: 0.59–0.86), 0.87 (95% CI: 0.78–0.96), and 0.93 (95% CI: 0.86–1.00), respectively. The fusion model demonstrated superior discriminative performance compared to the semantic and radiomics models across both cohorts.

**Figure 4:**
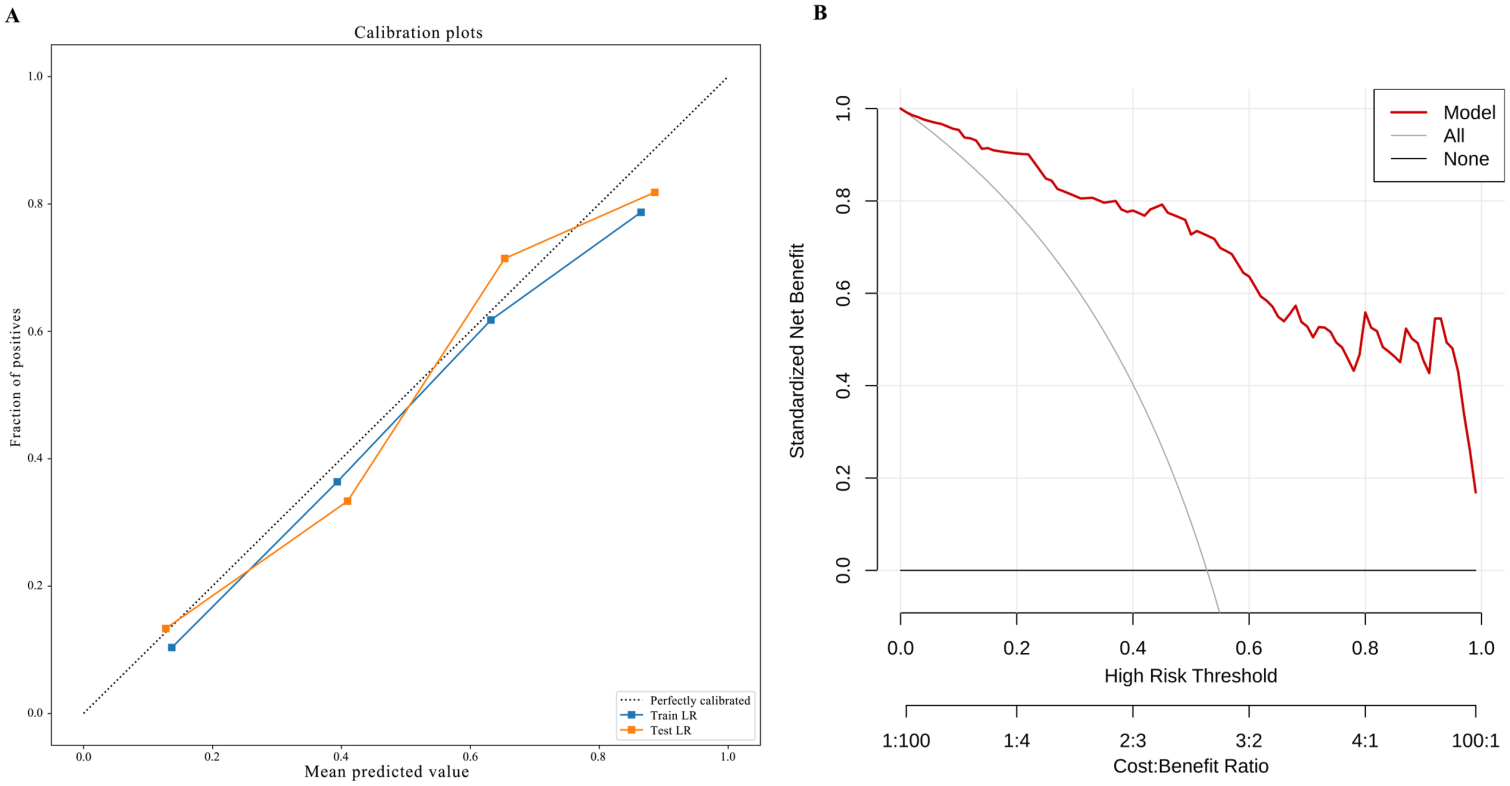
The calibration plot and decision curve of the fusion model. **(A)** Presents the calibration curve of the fusion model; **(B)** shows the decision curve for the fusion model. In calibration plot, with the *x*-axis representing the mean predicted values and the *y*-axis representing the fraction of positives. The gray line indicates perfect predictive performance under ideal conditions. The closer a model’s curve approximates the gray line, the better its predictive capability. In decision curve analysis, the *x*-axis represents the threshold probability and the *y*-axis represents net benefit. We report net benefit across a clinically meaningful range of thresholds rather than assuming a fixed threshold. A higher position of the model curve indicates greater clinical net benefit.

Figures 3A,B display the ROC curves of the three models in the training and test sets, respectively, while Table 3 and Supplementary Table S7 summarize performance metrics including AUC (95% CI), accuracy, sensitivity, specificity, F1 score, and Youden index in the training, test, and external validation cohorts. Classification metrics were calculated using the operating threshold that maximized the Youden index in the training set and were applied unchanged to the test and external validation sets. DeLong’s test revealed statistically significant differences (*p* < 0.05) in AUC between the fusion model and the other two models (Supplementary Table S8). Calibration curves and decision curves are presented in Figure 4 and Supplementary Figure S2, respectively. Ten-fold cross-validation within the training set supported the stability of all three models (Supplementary Figure S3).

**Table 3:** Predictive performance of the semantic, radiomics, and fusion models in the training and test cohorts.

Model	Train AUC (95% CI)	Train ACC	Train Sen	Train Spe	Train F1 score	Train Youden index	Test AUC (95%CI)	Test ACC	Test Sen	Test Spe	Test F1 score	Test Youden index
Semantic model	0.73 (0.64–0.81)	0.73	0.85	0.60	0.78	0.45	0.73 (0.59–0.86)	0.73	0.88	0.55	0.78	0.40
Radiomics model	0.84 (0.78–0.91)	0.80	0.80	0.79	0.81	0.59	0.87 (0.78–0.96)	0.84	0.82	0.86	0.85	0.68
Fusion model	0.94 (0.90–0.97)	0.88	0.91	0.84	0.89	0.75	0.93 (0.86–1)	0.87	0.94	0.80	0.89	0.74

AUC, area under the receiver operating characteristic curve; Acc, accuracy; Sen, sensitivity; Spe, specificity; CI, confidence interval; Youden, sensitivity + specificity − 1.

## Discussion

Aortic dissection is an acute, rapidly progressing, and highly fatal vascular emergency. Prompt diagnosis is challenging, as conventional assessment methods face significant limitations. Although CTA/MRA are often considered the “gold standard” imaging techniques for aortic dissection, they are invasive procedures involving radiation exposure and high costs, making them inappropriate for routine screening and long-term follow-up. Furthermore, clinical parameters and standard carotid ultrasound fail to detect subtle arterial wall abnormalities, which limits their utility for early warning and risk stratification in high-risk populations. Both the carotid artery and the aorta are elastic conduit vessels; sharing a highly similar embryonic origin, structure, and mechanical microenvironment, the microscopic degeneration of the carotid arterial wall can serve as a peripheral mirror marker for aortic wall vulnerability and susceptibility to dissection. Our study demonstrates that a carotid ultrasound radiomics model can help discriminate AD from non-AD participants. Integrating clinical data and ultrasound semantic features with Radscore to construct a fusion model improved discriminative performance, and external validation provided preliminary support for generalizability. Calibration assessment and decision curve analysis suggested potential clinical utility in triage contexts; Furthermore, the study seeks to elucidate the association between carotid artery pathologies and aortic diseases, offering a new technical pathway and research framework for the translation of scientific discoveries and the evaluation of therapeutic efficacy. However, prospective evaluation in the intended clinical workflow is required before any claims of risk prediction or prevention can be made.

Notably, as AD presentations may occur under acute physiological stress, elevations in SBP and other metabolic or inflammatory markers can reflect acute physiology and treatment context rather than pre-event baseline risk. Therefore, SBP should be interpreted primarily as a discriminative feature in the acute-presentation setting. Prospective pre-event cohorts are required to establish causal or preventive implications. This study successfully developed a nomogram that integrates carotid ultrasound radiomic features and clinical semantic characteristics. This model facilitates a noninvasive, individualized, and precise quantitative evaluation of AD risk, offering objective and reliable decision-making support for early screening, risk stratification, clinical intervention strategy development, and long-term follow-up management for high-risk populations.

Radiomics extracts high-throughput quantitative features from standard medical images (CT, MRI, PET, ultrasound) that are invisible to human visual assessment. These features are integrated with clinical, pathological, and genomic data to build diagnostic, prognostic, or predictive models (23). AI-based radiomics diagnostic/prediction models efficiently and objectively identify and analyze imaging features. They enhance lesion detection rates and diagnostic accuracy while reducing physician subjectivity and workload (24). Ultrasound radiomics extracts quantitative texture, grayscale, and morphological features from standard images, converting qualitative data into objective parameters to assess lesion heterogeneity (25). Applied in thyroid, breast, lymph node, and pelvic/abdominal masses, it improves diagnostic accuracy, risk stratification, and prognosis, enhancing non-invasive, precision diagnostics (26, 27).

The Darwin research platform was used for image upload, ROI annotation, and standardized radiomic feature extraction, which facilitated reproducible radiomics analysis without manual feature coding.

In our study, six radiomic features were ultimately selected after the initial 1,125 extracted features were reduced to 120 candidates through reproducibility assessment and feature-reduction procedures. These features were derived from second-order texture matrices (GLCM/GLDM) after exponential or square filtering and reflect lesion heterogeneity and gray-level dependency patterns.

Intima-media thickness (IMT) augmentation serves as an indicator of nascent atherosclerosis. Even within normative IMT parameters, nuanced structural alterations may manifest. In these instances, while sonographers cannot visually discern such modifications, radiomics exhibits its clinical utility—when incipient atherosclerotic transformations (such as lipid accumulation, microcalcifications, and heterogeneous fibrous hyperplasia) emerge within IMT zones: regional textural complexity intensifies, and DependenceEntropy elevates. This phenomenon potentially signifies preliminary manifestations of subclinical pathology (28). DCA addresses limitations of conventional metrics like AUC and accuracy by quantifying clinical net benefit rather than just predictive performance. It incorporates misclassification costs and risk preferences, visualizing model utility across decision thresholds to ensure patient benefit over statistical performance (29). Our fusion model demonstrates superior net benefit differential compared to baseline scenarios for AD identification in the studied setting.

Sample size was determined by the number of eligible participants available during the study period in this retrospective case–control design. To mitigate overfitting under limited sample size and relatively high feature dimensionality, we used reproducibility filtering, feature reduction and penalized selection (LASSO), internal resampling via 10-fold cross-validation, and independent external validation. Future multicenter studies with larger cohorts are warranted to further refine and prospectively evaluate the model.

Our study has certain limitations: 1. Owing to the retrospective observational design of our study, in which case enrollment and data acquisition relied on existing clinical records, it was inherently difficult to implement rigorous randomization and control. Consequently, the findings may be subject to selection bias, warranting further validation through prospective cohort studies. 2. While this study utilized external datasets for preliminary validation, the external cohort was limited in size. Future research should incorporate multi-institutional datasets from diverse regions to enhance generalizability and strengthen the robustness of the findings. 3. Given the low incidence of aortic dissection, the study’s sample size of 209 was constrained by recruitment challenges. To ensure rigor, stringent case selection and quality control were applied, excluding those with poor image quality or extensive missing data. Ten-fold cross-validation was employed to maximize data utility and mitigate overfitting. Model performance was assessed multi-dimensionally to enhance findings’ reliability. 4. In this study, only static two-dimensional images were selected for carotid ultrasound, without using dynamic videos, which may omit much useful information. Additionally, the elasticity index of carotid plaques was not included in the study, nor were hemodynamic changes considered. These are areas that need improvement in our subsequent research. 5. Since intraplaque hemorrhage is the best biological marker of plaque vulnerability (30, 31), it is also necessary to use contrast-enhanced ultrasound to assess intraplaque hemorrhage, which we will improve in subsequent studies. 6. In our study, the timing of data collection for carotid ultrasound and laboratory tests differed between patient groups. For acute aortic dissection patients, carotid ultrasound was completed within 12 h and laboratory tests within 8 h of admission, following hospital protocol. In contrast, no strict time window was set for non-aortic dissection patients; only results obtained within 1 week after the initial outpatient assessment were collected. This discrepancy in timing may introduce time-window bias between groups. In future research, consistency in the timing of data collection should be maintained across both patient groups to minimize potential bias and improve comparability and reliability of the findings. In addition, improving the reference standard for non-AD controls (e.g., broader use of CTA/MRA/TEE when clinically appropriate) may further reduce potential outcome misclassification in future work.

## Conclusion

This study utilized carotid ultrasound radiomics in conjunction with clinical and ultrasound semantic parameters to develop an integrated model that demonstrated robust performance in discriminating AD from non-AD participants in a retrospective dataset. The model may support clinical triage and hypothesis generation; prospective multicenter studies in the target population are required before making risk prediction or prevention claims.

## Data Availability

The raw data supporting the conclusions of this article will be made available by the authors, without undue reservation.

## Footnotes

1
https://pyradiomics.readthedocs.io/en/latest/features.html
