# Supplementary material for: Developing a carotid ultrasound radiomics-semantic fusion model to identify aortic dissection: a two-center retrospective study
Source: Front Med (Lausanne). 2026 Apr 29;13:1813428. doi: 10.3389/fmed.2026.1813428 (PMC13167551; doi:10.3389/fmed.2026.1813428)
Supplement: Supplementary file 1 [file Supplementary_File_1.DOCX]

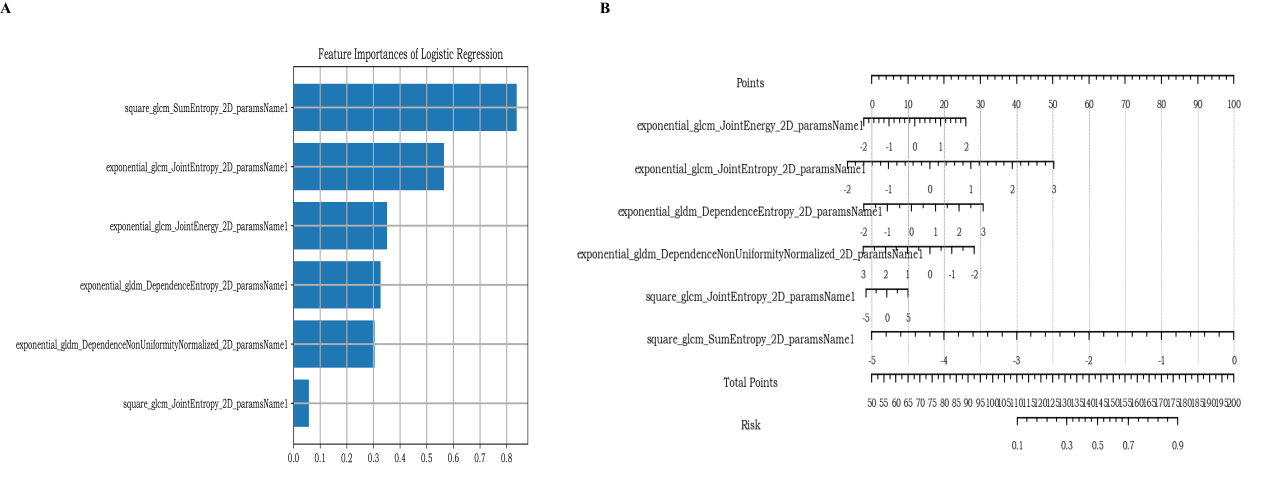


Figure S1. Visualization of the six radiomic features selected by LASSO and the corresponding radiomics model. (A) Relative coefficients/weights of the six selected radiomic features. (B) Nomogram of the radiomics model constructed from these six features.


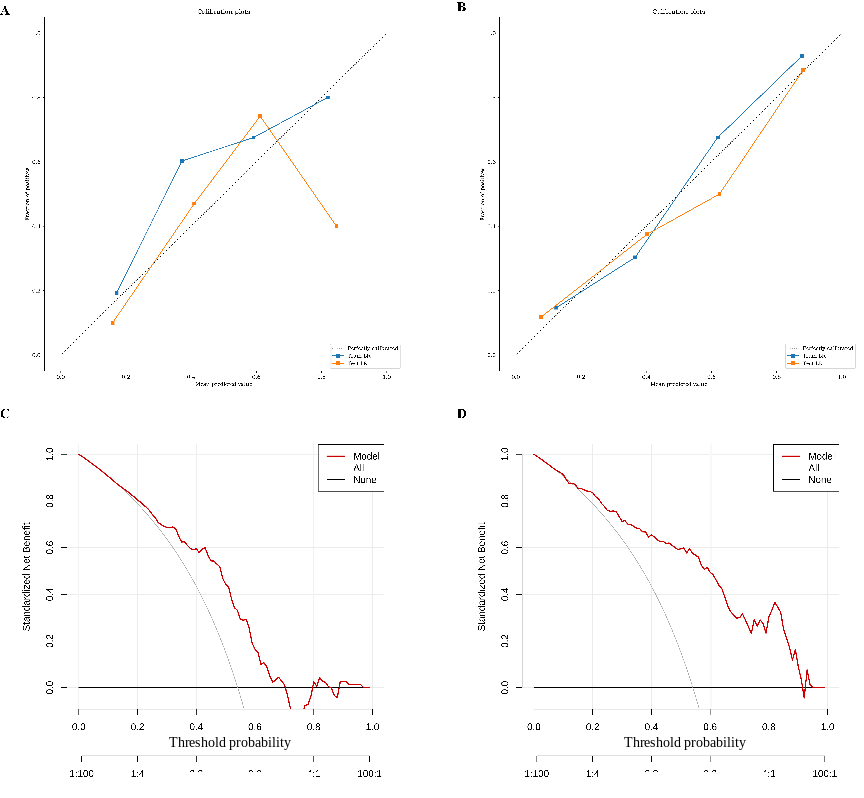


Figure S2. Calibration curves and decision curve analysis (DCA) for the three models. (A) Calibration curves in the internal cohort. (B) Calibration curves in the external validation cohort. (C) DCA in the internal cohort. (D) DCA in the external validation cohort. In DCA, the x-axis represents the threshold probability (a secondary axis may show the equivalent cost–benefit ratio), and the y-axis represents net benefit. A model provides clinical utility over a range of thresholds when its net benefit curve lies above the ‘treat-all’ and ‘treat-none’ strategies.


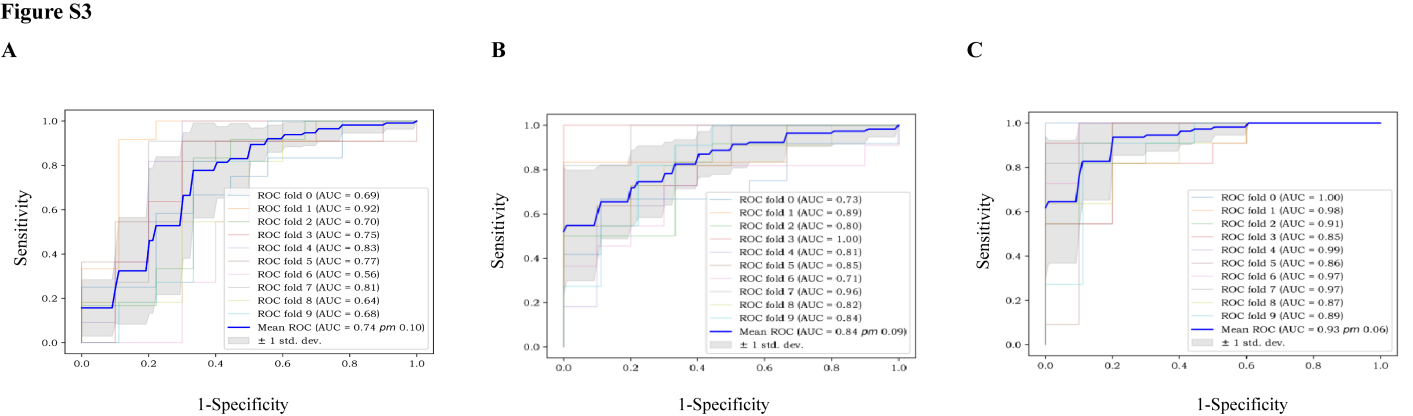


Figure S3. Ten-fold cross-validation ROC curves of the three models in the training set. The horizontal axis represents 1 − specificity and the vertical axis represents sensitivity. The cross-validated AUCs for the semantic, radiomics, and fusion models were 0.74, 0.84, and 0.93, respectively.

**Methods for handling missing values:**

Missing values were observed in several laboratory markers (e.g., homocysteine, creatinine, SBP/DBP, A/G, lipid profile, blood glucose, and ALT/AST). The number and proportion of missing values are summarized in Supplementary Tables S1–S2. Because missingness was <10% for all variables, we applied single imputation to preserve sample size for modelling.

Imputation was performed using the training set only: continuous variables were imputed using the mean (approximately normal) or median (skewed), and categorical variables using the mode. The fitted imputation parameters were then applied unchanged to the test and external validation cohorts (i.e., without refitting on those datasets).

Table S1. Missingness summary for clinical variables in the internal cohort (n=209).

| Variable | Missing count | Missing proportion (%) |
| --- | --- | --- |
| Homocysteine | 18 | 8.6 |
| Creatinine | 13 | 6.2 |
| SBP | 9 | 4.3 |
| DBP | 9 | 4.3 |
| A/G | 15 | 7.2 |
| TC | 12 | 5.7 |
| HDL | 12 | 5.7 |
| LDL | 12 | 5.7 |
| Blood glucose | 7 | 3.3 |
| ALT/AST | 8 | 3.8 |

**Table S2. Missingness summary for clinical variables in the external validation cohort (n=39).**

| Variable | Missing count | Missing proportion (%) |
| --- | --- | --- |
| Homocysteine | 3 | 7.7 |
| Creatinine | 2 | 5.1 |
| SBP | 1 | 2.6 |
| DBP | 1 | 2.6 |
| A/G | 2 | 5.1 |
| TC | 3 | 7.7 |
| HDL | 3 | 7.7 |
| LDL | 3 | 7.7 |
| Blood glucose | 1 | 2.6 |
| Uric acid | 2 | 5.1 |
| ALT/AST | 2 | 5.1 |

Table S3. Intra- and inter-observer agreement of ultrasound semantic measurements assessed by intraclass correlation coefficients (ICC).

|  | Plaque Echogenicity | Number of plaques | IMT | Smoothness of plaque | Smoothness of carotid inter-media | Maximum plaque thickness |
| --- | --- | --- | --- | --- | --- | --- |
| ICC | 0.94 | 0.96 | 0.89 | 0.91 | 0.93 | 0.92 |
| 95%CI | (0.93-0.95) | (0.94-0.97) | (0.86-0.91) | (0.88-0.93) | (0.91-0.95) | (0.90-0.94) |

ICC: intra-group correlation coefficient. CI: confidence interval

**Table S4. Participants’ demographic and clinical characteristics in the external validation cohort.**

| Characteristic | AD  (n=18) | Non-AD  (n=21) | p |
| --- | --- | --- | --- |
| Age | 62.28±8.86 | 59.71±9.94 | 0.40a |
| Gender |  |  | 0.34b |
| Female | 5 | 9 |  |
| Male | 13 | 12 |  |
| BMI(Kg/m2) | 26.25±3.24 | 25.44±2.18 | 0.36a |
| Plaque Echogenicity |  |  | 0.24b |
| Hypoechoic plaque | 5 | 3 |  |
| Predominantly hypoechoic heterogeneous plaque | 5 | 6 |  |
| Predominantly hyperechoic heterogeneous plaque | 6 | 7 |  |
| Hyperechoic plaque | 2 | 5 |  |
| Number of plaques |  |  | 0.29b |
| Solitary plaque | 10 | 8 |  |
| Multiple plaques | 8 | 13 |  |
| IMT(mm) | 1.03±0.27 | 1.01±0.26 |  |
| Smoothness of plaque |  |  | 0.10b |
| Smooth | 6 | 6 |  |
| Non-smooth | 12 | 15 |  |
| Smoothness of carotid inter-media |  |  | 0.45b |
| Smooth | 7 | 6 |  |
| Non-smooth | 11 | 15 |  |
| Maximum plaque thickness | 2.63±0.73 | 2.38±0.52 | 0.22a |
| Lymphocyte (×109/L) | 1.44±0.85 | 1.56±0.61 | 0.61a |
| SBP(mmHg) | 194.44±82 | 162.57±12.30 | 0.01a |
| DBP(mmHg) | 103.33±19.01 | 89.19±11.10 | <0.001a |
| WBC (×109/L) | 11.26±2.84 | 8.02±3.71 | 0.01a |
| HDL(mmol/L) | 0.97±0.26 | 1.11±0.30 | 0.14a |
| LDL(mmol/L) | 2.62±0.74 | 2.50±1.22 | 0.73a |
| TC (mmol/L) | 4.56±0.83 | 4.24±1.37 | 0.40a |
| RC (mmol/L) | 0.98±0.27 | 0.64±0.14 | <0.001a |
| Uric acid(μmol/L) | 315.39±183.30 | 364.05±184.00 | 0.42a |
| Blood glucose (mmol/L) | 7.77±2.36 | 6.45±1.93 | 0.06a |
| Creatinine (μmol/L) | 77.13(36.22) | 78.62(37.01) | 0.89a |
| Homocysteine(μmol/L) | 13.41±2.80 | 15.87±5.17 | 0.08a |
| ALT/AST | 0.92±0.32 | 1.07±0.50 | 0.26a |
| A/G | 1.33±0.29 | 1.31±0.34 | 0.90a |

a One-way ANOVA or Kruskal-Wallis test (as appropriate)

b Chi-square test or Fisher's exact test (as appropriate)

AD means Aortic dissection, BMI means Body mass index, SBP means Systolic blood pressure, DBP means Diastolic blood pressure, WBC means White blood cell, HDL means High-density lipoprotein, LDL means Low-density lipoprotein, TC means Total cholesterol, RC means Remnant cholesterol, ALT means Alanine Aminotransferase, AST means Aspartate Aminotransferase, A/G means  Albumin/Globulin Ratio.

**Table S5. Baseline comparability across the training, test, and external validation cohorts.**

| Characteristic | Training set  (n=146) | Test set  (n=63) | External validation set(n=39) | p |
| --- | --- | --- | --- | --- |
| Age(years old) | 62.28±13.72 | 59.81±14.92 | 60.90±9.42 | 0.46a |
| Gender |  |  |  | 0.96b |
| Female | 56 | 24 | 14 |  |
| Male | 90 | 39 | 25 |  |
| Label |  |  |  | >0.99b |
| AD | 68 | 29 | 18 |  |
| Non-AD | 78 | 34 | 21 |  |
| Plaque echogenicity |  |  |  | 0.93b |
| Hypoechoic plaque | 32 | 13 | 8 |  |
| Predominantly hypoechoic heterogeneous plaque | 50 | 17 | 11 |  |
| Predominantly hyperechoic heterogeneous plaque | 39 | 21 | 13 |  |
| Hyperechoic plaque | 25 | 12 | 7 |  |
| Number of plaques |  |  |  | 0.77b |
| Solitary plaque | 64 | 31 | 18 |  |
| Multiple plaques | 82 | 32 | 21 |  |
| IMT(mm) | 1.13±0.23 | 1.13±0.23 | 1.12±0.24 | 0.93a |
| Smoothness of plaque |  |  |  | 0.24b |
| Smooth | 82 | 30 | 12 |  |
| Non-smooth | 64 | 33 | 27 |  |
| Smoothness of carotid  inter-media |  |  |  | 0.42b |
| Smooth | 53 | 20 | 13 |  |
| Non-smooth | 93 | 43 | 26 |  |
| Maximum plaque thickness | 2.47±0.82 | 2.35±0.96 | 2.49±0.43 | 0.33a |
| Lymphocyte (×109/L) | 1.47±0.67 | 1.37±0.60 | 1.15±0.72 | 0.50a |
| BMI(Kg/m2) | 25.98±3.46 | 25.79±3.61 | 25.81±0.71 | 0.97a |
| SBP(mmHg) | 173.25±27.33 | 168.52±25.69 | 177.28±23.49 | 0.25a |
| DBP(mmHg) | 94.51±18.17 | 93.73±18.37 | 95.72±16.65 | 0.86a |
| WBC (×109/L) | 9.32±3.59 | 8.89±3.02 | 9.51±3.68 | 0.62a |
| HDL(mmol/L) | 1.05±0.23 | 1.02±0.27 | 1.04±0.29 | 0.77a |
| LDL(mmol/L) | 2.72±0.89 | 2.76±0.79 | 2.26±1.02 | 0.51a |
| TC (mmol/L) | 4.55±0.98 | 4.46±0.87 | 4.39±1.15 | 0.63a |
| RC (mmol/L) | 0.76±0.34 | 0.68±0.33 | 0.79±0.27 | 0.10a |
| Uric acid(μmol/L) | 328.69±119.98 | 379.27±148.66 | 341.59±182.90 | 0.56a |
| Blood glucose (mmol/L) | 7.20±2.41 | 7.08±2.61 | 7.06±2.21 | 0.93a |
| Creatinine (μmol/L) | 70.22(33.28) | 76.14(29.82) | 73.26(26.70) | 0.90a |
| Homocysteine(μmol/L) | 14.95±8.30 | 14.94±6.81 | 14.73±4.37 | 0.99a |
| ALT/AST | 1.03±0.42 | 0.97±0.39 | 0.99±0.43 | 0.65a |
| A/G | 1.33±0.32 | 1.37±0.32 | 1.32±0.31 | 0.72a |

a One-way ANOVA or Kruskal-Wallis test (as appropriate)

b Chi-square test or Fisher's exact test (as appropriate)

AD means Aortic dissection, BMI means Body mass index, SBP means Systolic blood pressure, DBP means Diastolic blood pressure, WBC means White blood cell, HDL means High-density lipoprotein, LDL means Low-density lipoprotein, TC means Total cholesterol, RC means Remnant cholesterol, ALT means Alanine Aminotransferase, AST means Aspartate Aminotransferase, A/G means  Albumin/Globulin Ratio.

**Table S6. Univariate and multivariable logistic regression analyses based on ultrasound semantic and clinical variables.**

| Variable | Univariate OR (95% CI) | P value | Multivariable OR (95% CI) | P value |
| --- | --- | --- | --- | --- |
| Age | / | 0.69 | / | / |
| Gender | / | 0.70 | / | / |
| Plaque echogenicity | 3.62 (2.48–5.27) | <0.001 | 2.22 (1.36–3.11) | 0.03 |
| Number of plaques | / | 0.68 | / | / |
| IMT (mm) | / | 0.33 | / | / |
| Smoothness of plaque | / | 0.67 | / | / |
| Smoothness of carotid intima–media | / | 0.16 | / | / |
| Maximum plaque thickness (mm) | 0.59 (0.42–0.83) | 0.003 | / | 0.55 |
| BMI (kg/m²) | / | 0.62 | / | / |
| SBP (mmHg) | 1.32 (0.89–0.94) | <0.001 | 0.96 (0.93–0.98) | 0.002 |
| DBP (mmHg) | 1.03 (0.91–0.95) | <0.001 | / | 0.34 |
| WBC | 0.70 (0.63–0.78) | <0.001 | / | 0.88 |
| HDL (mmol/L) | 5.57 (1.69–18.40) | 0.005 | / | 0.42 |
| LDL (mmol/L) | 0.01 (0.002–0.03) | 0.14 | / | 0.54 |
| TC (mmol/L) | 0.64 (0.47–0.87) | 0.005 | / | / |
| RC (mmol/L) | 3.71 (1.01–6.22) | <0.001 | 0.07 (0.01–0.52) | 0.009 |
| Uric acid (μmol/L) | / | 0.40 | / | / |
| Blood glucose (mmol/L) | 0.76 (0.66–0.88) | <0.001 | / | 0.48 |
| Creatinine (μmol/L) | / | 0.87 | / | / |
| Homocysteine (μmol/L) | / | 0.11 | / | / |
| ALT/AST | / | 0.77 | / | / |
| A/G | 7.29 (2.81–18.89) | <0.001 | / | 0.58 |
| Radscore | 2.81 (2.12–3.72) | <0.001 | 2.36 (1.40–3.98) | 0.001 |
| Lymphocyte count | 3.11 (1.48–6.55) | <0.001 | / | 0.08 |

Note: '/' indicates that the estimate was not available or the variable was not retained in the multivariable model.

AD means Aortic dissection, BMI means Body mass index, SBP means Systolic blood pressure, DBP means Diastolic blood pressure, WBC means White blood cell, HDL means High-density lipoprotein, LDL means Low-density lipoprotein, TC means Total cholesterol, RC means Remnant cholesterol, ALT means Alanine Aminotransferase,AST means Aspartate Aminotransferase, A/G means  Albumin/Globulin Ratio.

**Table S7. Predictive performance of the three models in the external validation cohort.**

| Model | External AUC  (95%CI) | External  ACC | External  Sen | External  Spe | External  F1 Score | External  Youden index |
| --- | --- | --- | --- | --- | --- | --- |
| Semantic model | 0.71(0.58-0.85) | 0.75 | 0.94 | 0.52 | 0.80 | 0.46 |
| Radiomics model | 0.81(0.73-0.88) | 0.77 | 0.83 | 0.70 | 0.80 | 0.53 |
| Fusion model | 0.91(0.83-1) | 0.88 | 0.89 | 0.89 | 0.90 | 0.78 |

AUC, area under the receiver operating characteristic curve; Acc, accuracy; Sen, sensitivity; Spe, specificity; CI, confidence interval; Youden, sensitivity + specificity - 1.

Table S8. DeLong tests comparing AUCs between models in the training, test, and external validation cohorts.

| Model Comparison | Training set (n=146) | | Test set (n=63) | |
| --- | --- | --- | --- | --- |
|  | Z | P | Z | P |
| Semantic model - Fusion model | 3.69 | <0.001 | 3.21 | <0.001 |
| Radiomics model - Fusion model | 2.42 | 0.02 | 1.97 | 0.04 |
| Semantic model- Radiomics model | 2.56 | 0.004 | 2.34 | 0.01 |

**The procedure for ROI segmentation and sample image presentation:**

Define the region of interest (ROI). High-resolution 2D carotid ultrasound images (left and right) were uploaded to the Darwin research platform. For radiomics analysis, one representative image per participant was selected from the side showing the most pronounced atherosclerotic change (largest plaque area, or the greatest IMT thickening/irregularity when no plaque was present). If both sides were comparable, the right side was selected. The ROI was manually delineated using the freehand tool: for images with plaques, the ROI encompassed the entire plaque on the frame with maximal plaque area, excluding the lumen and acoustic shadow; for images without plaque, the ROI covered a 1-cm distal common carotid artery segment proximal to the bifurcation including the intima and media layers. ROIs were annotated by trained readers blinded to outcomes, and radiomic features were automatically extracted using PyRadiomics implemented in the platform (including texture features such as GLCM and GLDM from original and filtered images, e.g., square and exponential). An example ROI annotation is shown in Figure S4.

Figure S4. Example of ROI delineation on carotid ultrasound images used for radiomic feature extraction.


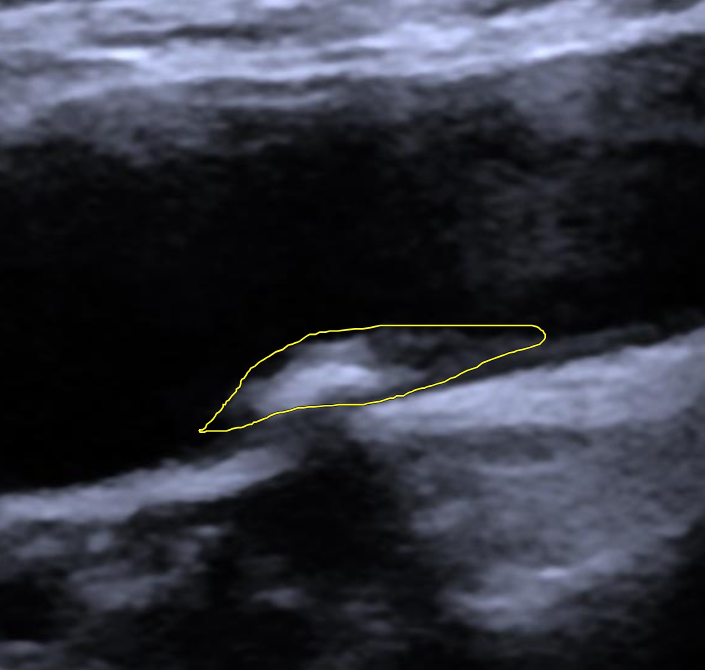


After ROI annotation, the platform exports the ROI mask and the radiomic feature matrix for downstream statistical analysis.

**Table S9. Software and package versions used in this study.**

| Software | Version |
| --- | --- |
| PyRadiomics | 2.0 |
| rms | 8.1-0 |
| rmda | 2.0.0 |
| riskRegression | 2023.09.26 |
| pROC | 1.19.0 |
| glmnet | 4.1-10 |
| mice | 2.46.0 |
| MedCalc | 23.4.8 |
| R | 4.3.3 |
| Darwin research platform | 2.0 |

All images were uploaded and annotated using the Darwin research platform, which integrates PyRadiomics for standardized radiomic feature extraction.

**Table S10. Mapping of radiomic feature names between the Darwin/PyRadiomics export and the simplified names used in the manuscript.**

| Exported feature name | Name used in manuscript | Notes |
| --- | --- | --- |
| square_glcm_SumEntropy_2D_paramsName1 | square_glcm_SumEntropy | Square-filtered image; GLCM texture feature |
| exponential_glcm_JointEntropy_2D_paramsName1 | exponential_glcm_JointEntropy | Exponential-filtered image; GLCM texture feature |
| exponential_glcm_JointEnergy_2D_paramsName1 | exponential_glcm_JointEnergy | Exponential-filtered image; GLCM texture feature |
| exponential_gldm_DependenceEntropy_2D_paramsName1 | exponential_gldm_DependenceEntropy | Exponential-filtered image; GLDM texture feature |
| exponential_gldm_DependenceNonUniformityNormalized_2D_paramsName1 | exponential_gldm_DependenceNonUniformityNormalized | Exponential-filtered image; GLDM texture feature |
| square_glcm_JointEntropy_2D_paramsName1 | square_glcm_JointEntropy | Square-filtered image; GLCM texture feature |
